# When Hypercapnia Misleads: A Case of Respiratory-Onset Motor Neuron Disease

**DOI:** 10.7759/cureus.117375

**Published:** 2026-10-03

**Authors:** Yousef Kordi, Mohamed Ismail, Elizabeth Estabrook

**Affiliations:** 1 Gastroenterology, University Hospitals Dorset NHS Foundation Trust, Poole, GBR; 2 Acute Medicine, University Hospital Southampton NHS Foundation Trust, Southampton, GBR; 3 General Medicine, University Hospital Southampton NHS Foundation Trust, Southampton, GBR

**Keywords:** anchoring bias, diagnostic delay, hypercapnic respiratory failure, motor neuron disease, neuromuscular hypoventilation, non-invasive ventilation, respiratory-onset mnd

## Abstract

Motor neuron disease (MND) most commonly presents with limb or bulbar weakness; however, a minority of patients present with respiratory-onset disease, posing significant diagnostic challenges. We report the case of a 78-year-old woman with a background of small airways disease and rheumatoid arthritis not requiring treatment for many years, who presented with a two-year history of progressive exertional dyspnoea, unintentional weight loss, and functional decline. Before diagnosis, she underwent extensive investigation across primary and secondary care, including chest radiography, spirometry, computed tomography imaging, and ear, nose, and throat specialist assessment, without a unifying diagnosis being established. On admission, venous blood gas demonstrated compensated hypercapnia, which was attributed to obstructive airways disease. She subsequently developed hypercapnic respiratory failure, and a clinical diagnosis of MND was made on the basis of generalised muscle wasting, fasciculations, brisk lower-limb reflexes, and electromyography findings. Non-invasive ventilation was initiated with significant clinical improvement. This case highlights the diagnostic challenges of respiratory-onset MND, the risk of anchoring bias in the interpretation of biochemical markers, and the importance of considering neuromuscular causes when respiratory symptoms are disproportionate to radiological and biochemical findings.

## Introduction

Motor neuron disease (MND) is a progressive neurodegenerative disorder characterised by degeneration of upper and lower motor neurons, resulting in progressive muscle weakness and, in many patients, respiratory muscle weakness and respiratory failure. Amyotrophic lateral sclerosis is the most common form of MND, though other phenotypes, including progressive muscular atrophy (PMA), are recognised. Median survival from symptom onset is approximately three years, and respiratory failure is the most common cause of death [1,2]. 

MND has heterogeneous patterns of onset, most commonly involving limb or bulbar muscles. A small proportion of patients present with respiratory muscle weakness as the predominant initial manifestation of disease, before clinically apparent limb or bulbar weakness [1]. Respiratory-onset MND is rare, accounting for approximately 1%-4% of presentations, and may manifest with progressive dyspnoea, orthopnoea, sleep-related hypoventilation, and fatigue [3-5]. Respiratory muscle weakness reduces tidal volume and impairs the ability to increase minute ventilation in response to rising carbon dioxide, leading first to nocturnal hypoventilation and, as compensatory mechanisms fail, to chronic daytime hypercapnia with a compensatory rise in bicarbonate [1]. 

Diagnostic delay in MND averages 10-16 months from symptom onset [1]. Atypical presentations, including respiratory-onset disease, can contribute to diagnostic delay because initial symptoms may mimic more common cardiopulmonary disorders [3,5]. Unintentional weight loss may occur in MND because of muscle wasting, reduced nutritional intake, and increased metabolic demand, and when prominent, may lead clinicians to initially investigate malignancy or systemic disease [3].

This case illustrates the diagnostic challenges of respiratory-onset MND and highlights how attribution of hypercapnia to coexisting obstructive airway disease may delay recognition of neuromuscular respiratory failure.

## Case presentation

A 78-year-old woman was referred to the acute medical team in May 2025 with a two-year history of progressive functional decline characterised by exertional breathlessness, fatigue, generalised weakness, and severe unintentional weight loss from 75 kg to 45 kg (body mass index: 18.7 kg/m²). She also reported reduced oral intake, which she attributed to a change in taste following a suspected COVID-19 infection in 2019, although testing was unavailable at the time. Four months before admission, she experienced rapid functional deterioration with loss of appetite and significant further weight loss. 

Before admission, the patient underwent extensive investigation for respiratory symptoms and fluctuating weight loss without a clear unifying diagnosis. 

In 2023, she presented to her general practitioner as a lifelong non-smoker with exertional dyspnoea and productive cough. Chest radiography demonstrated large lung volumes. Spirometry demonstrated an obstructive pattern. Computed tomography of the chest, abdomen, and pelvis (CT CAP) showed bilateral pulmonary nodules up to 5 mm and mild bronchiolitis with mucous plugging; interval CT surveillance was arranged in accordance with British Thoracic Society (BTS) guidance for incidentally detected pulmonary nodules, and she was subsequently discharged from surveillance after the nodules remained stable. 

At respiratory review in February 2024, formal pulmonary function testing confirmed significant obstruction (forced expiratory volume in one second (FEV₁): 1.06 L; forced vital capacity (FVC): 1.70 L; FEV₁/FVC: 62%; fractional exhaled nitric oxide: normal). Static lung volumes by body plethysmography demonstrated a preserved total lung capacity (TLC: 4.86 L) with an elevated residual volume (RV: 3.15 L) and RV/TLC ratio (65%), in keeping with gas trapping and hyperinflation rather than a restrictive process; the reduced vital capacity was therefore attributed to air trapping in the context of obstructive disease. A long-acting beta-agonist was commenced for suspected small airways disease. Concurrent changes in vocal quality and globus sensation were investigated by ENT; flexible nasoendoscopy excluded structural upper airway pathology. At review in May 2024, bedside spirometry demonstrated significant improvement (FEV₁: 1.54 L, 65% predicted; FVC: 2.56 L, 82% predicted), both symptoms had resolved, and she was discharged from respiratory care on a long-acting bronchodilator. She was referred for pulmonary rehabilitation, which she was unable to attend.

In late 2024, she developed a four-month history of progressive atraumatic neck pain, worse on the right side, evolving into difficulty supporting the weight of her head. This was accompanied by marked functional decline (no longer able to walk or swim far, or garden for any length of time), anorexia, and further weight loss. Cervical spine radiography demonstrated facet joint disease and disc height loss. She was referred for orthopaedic review and magnetic resonance imaging (MRI) of the cervical spine; however, this had not been performed by the time of her subsequent hospital admission with progressive functional decline and dyspnoea. 

On admission, she appeared cachectic but had stable vital signs with oxygen saturation of 97% on room air. VBG demonstrated compensated hypercapnia (pH: 7.46, partial pressure of carbon dioxide (pCO₂): 7.45 kPa, bicarbonate (HCO₃⁻): 39.4 mmol/L; reference range: 22-26 mmol/L), initially attributed to obstructive airway disease. Chest radiography showed hyperinflated lung fields without focal consolidation (Figure 1). Routine blood tests, including full blood count, renal profile, bone profile, liver profile, C-reactive protein, coagulation screen, and blood-borne virus screening were unremarkable, with the exception of hyponatraemia (sodium: 126 mmol/L); adrenal and thyroid disease were subsequently excluded. 

**Figure 1:**
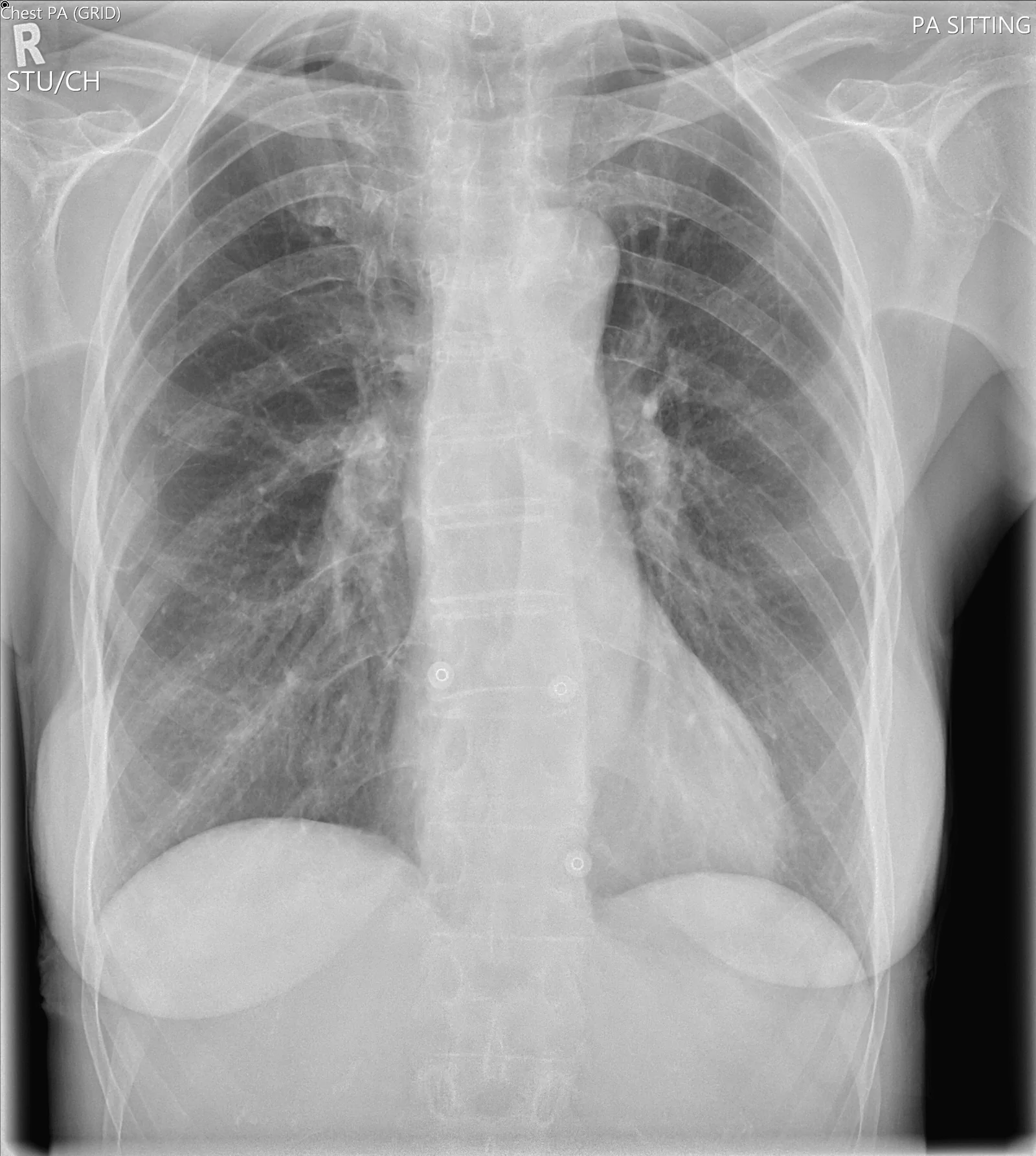
Posterior-anterior chest radiograph on admission demonstrating hyperinflated lung fields with coarse bronchovascular markings. No consolidation, mass, pleural effusion, or bony lesion identified. Cardiomediastinal contours are preserved.

Given the severity of weight loss, extensive investigations were performed. CT CAP demonstrated no evidence of malignancy, and upper gastrointestinal endoscopy was unremarkable. Coeliac disease was excluded. Psychiatric assessment concluded that an eating disorder was not contributing to her anorexia. Enteral feeding was commenced for nutritional support. 

During admission, she developed an episode of hypoventilation with worsening hypercapnia and reduced conscious level. Neurological examination revealed generalised muscle wasting, fasciculations, and brisk lower-limb reflexes, raising clinical suspicion for MND. Spirometry demonstrated a markedly reduced FVC of approximately 0.73 L, prompting serial FVC monitoring and urgent neurology referral. Formal respiratory muscle testing, such as sniff nasal inspiratory pressure or maximal inspiratory and expiratory pressures, was not performed; serial FVC was used to monitor respiratory function. She subsequently experienced acute deterioration with worsening hypercapnic respiratory failure on arterial blood gas (ABG) analysis (pH: 7.15, pCO₂: 16.5 kPa, HCO₃⁻: 43.3 mmol/L), and non-invasive ventilation (NIV) was initiated. 

Electromyography (EMG) and nerve conduction studies performed during the inpatient admission excluded a neuromuscular junction disorder, myopathy, and peripheral neuropathy as contributing causes. The findings were felt to be in keeping with an anterior horn cell disorder, with MND considered possible, though the electrophysiologist noted an atypical clinical phenotype and suggested a possible PMA phenotype. Clinical assessment nonetheless demonstrated combined upper and lower motor neuron involvement throughout, supporting a diagnosis of MND. Repeat EMG performed at outpatient neurology review in January 2026 demonstrated modest electrophysiological progression and was reported as supportive of a diagnosis of MND. MRI of the cervical spine demonstrated multilevel cervical spondylosis (C3-C7), with degenerative disc-osteophyte bars causing moderate thecal sac narrowing at C3/4. The spinal cord returned normal signal with no evidence of cord compression, excluding a compressive myelopathy as the cause of her neurological presentation (Figures 2, 3). Pompe disease was excluded on the basis of a normal acid alpha-glucosidase enzyme assay.

**Figure 2:**
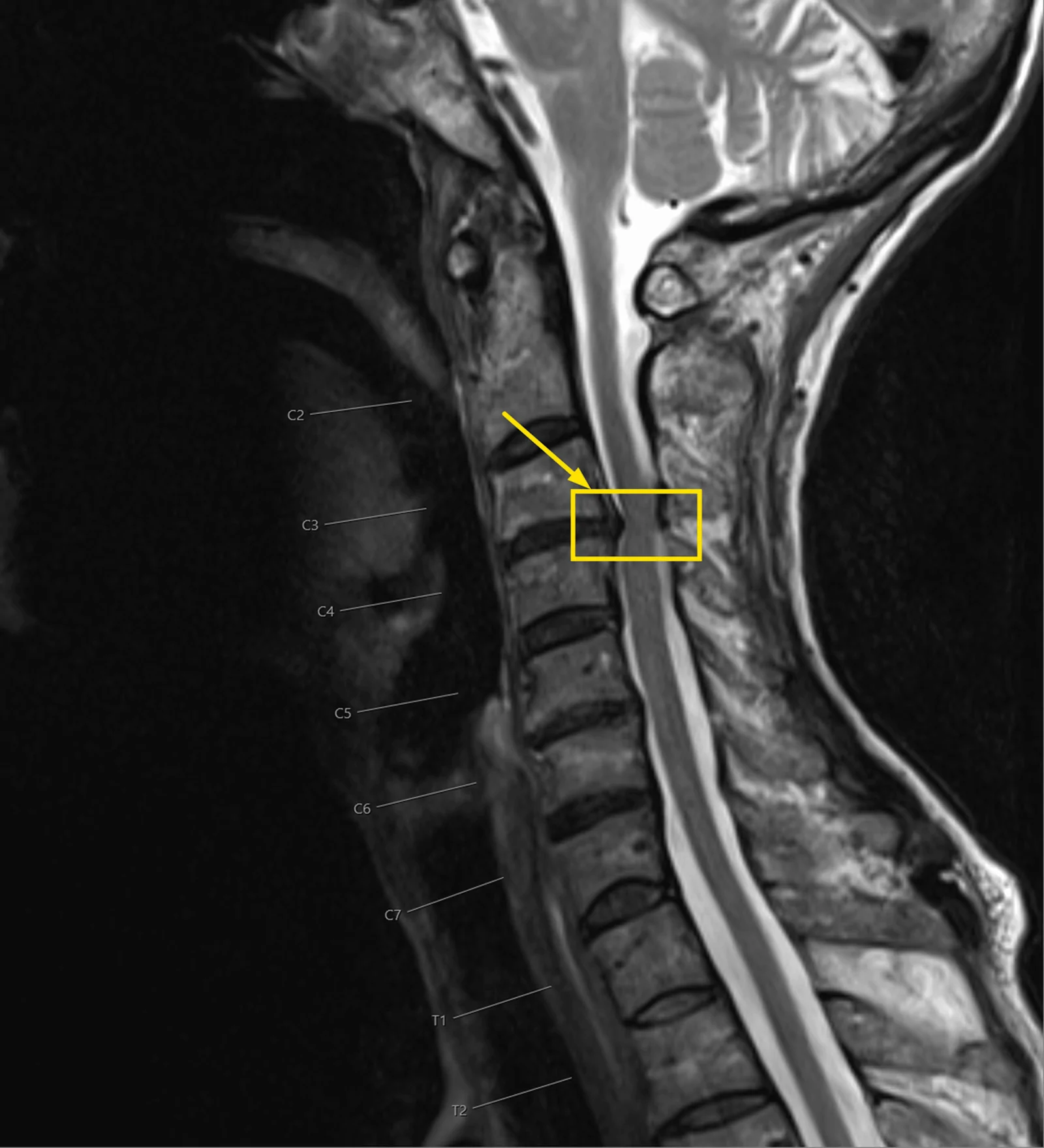
Sagittal T2-weighted MRI of the cervical spine (C2-T2) demonstrating reversal of the normal cervical lordosis with degenerative disc-osteophyte complexes from C3-C7. The yellow box highlights moderate thecal sac narrowing at C3/4 (arrow), where the anterior disc-osteophyte complex indents the ventral thecal sac and reduces the surrounding CSF space without clear cord compression. The cervical spinal cord demonstrates normal signal throughout, and the craniocervical junction is preserved.

**Figure 3:**
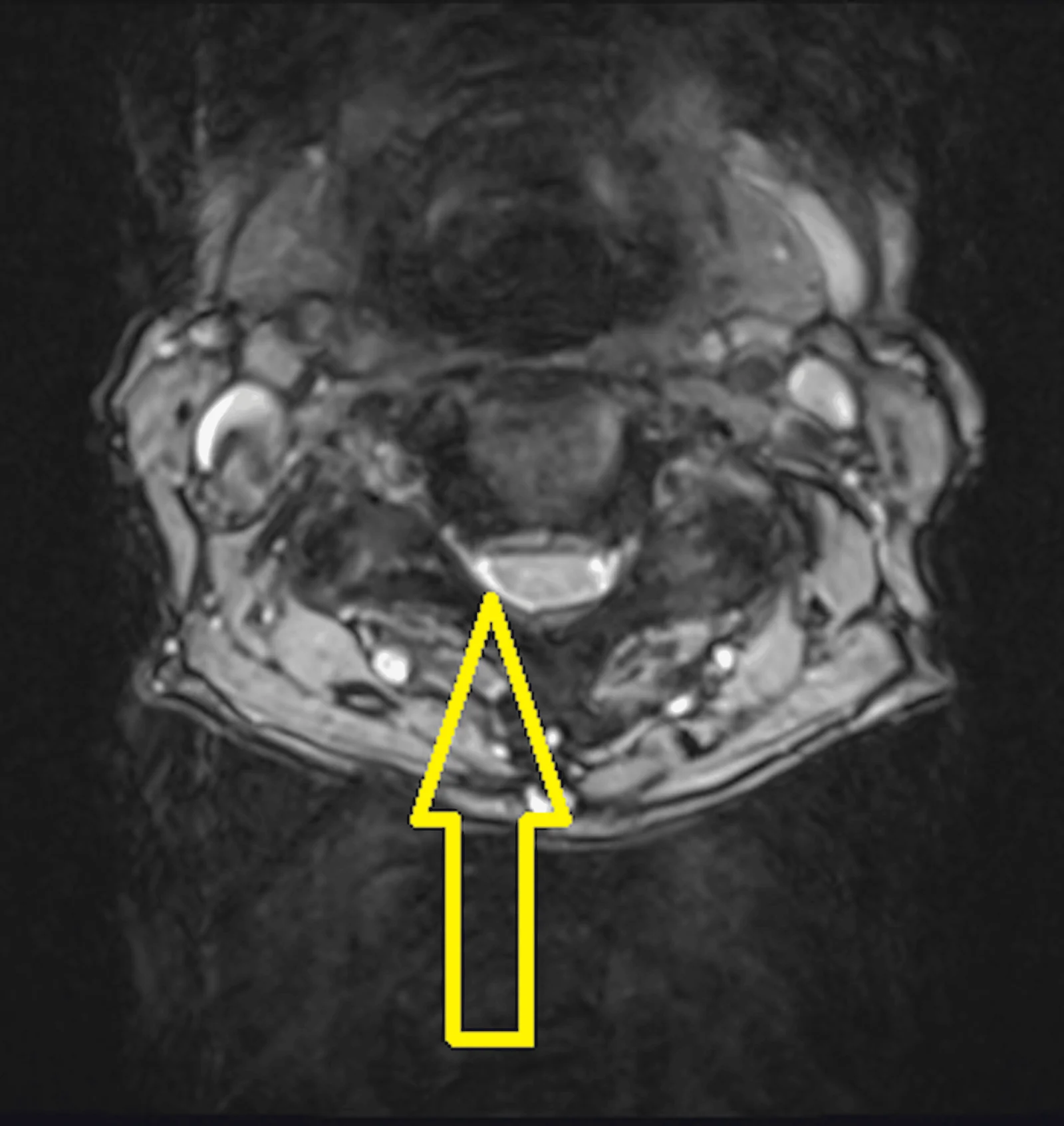
Axial T2-weighted MRI at the C3/4 level demonstrating moderate thecal sac narrowing. The arrow indicates the anterior disc-osteophyte complex, which indents the ventral thecal sac and reduces the surrounding CSF space without clear spinal cord compression, corresponding to the sagittal finding in Figure 2.

Given the clinical features of respiratory failure, widespread fasciculations, and upper motor neuron signs, a clinical diagnosis of MND was made. Following initiation of NIV, there was a rapid response, with improvement in hypercapnia and functional status within 24-48 hours. The patient stabilised on nocturnal NIV and was discharged with community neurorehabilitation follow-up. At outpatient review, ABG analysis demonstrated improved ventilation (pH: 7.42, pCO₂: 5.41 kPa, HCO₃⁻: 26.3 mmol/L) (Table 1). 

**Table 1:** Serial blood gas parameters demonstrating progressive hypercapnic respiratory failure with compensatory metabolic alkalosis, and subsequent normalisation following initiation of non-invasive ventilation. ABG: arterial blood gas; HCO₃⁻: bicarbonate; pCO₂: partial pressure of carbon dioxide; VBG: venous blood gas.

Parameter	Reference range	On admission (VBG)	Acute deterioration (ABG)	Outpatient follow-up (ABG)
pH	7.35 -7.45	7.457	7.154	7.42
pCO₂ (kPa)	4.7-6.0	7.46	16.5	5.41
HCO₃⁻ (mmol/L)	22-26	39.4	43.3	26.3

## Discussion

Respiratory-onset MND

Respiratory involvement in MND usually occurs later in the disease course; however, respiratory-onset disease is a recognised phenotype accounting for approximately 1%-4% of cases [3-5]. Patients may present with dyspnoea, fatigue, or sleep-related hypoventilation before limb weakness becomes clinically evident, and this atypical sequence frequently results in extensive cardiopulmonary investigation before a neuromuscular aetiology is considered [6]. 

In the present case, early symptoms of weight loss, fatigue, and progressive breathlessness directed investigation towards systemic and malignant causes over a prolonged period. Weight loss is frequently observed in MND due to muscle wasting, dysphagia, and hypermetabolism, but it is rarely the dominant presenting feature and may therefore obscure the diagnosis [6]. The degree of respiratory compromise and the eventual acute hypercapnic decompensation were disproportionate to both her pulmonary function and imaging findings, a pattern suggestive of a neuromuscular aetiology.

Cognitive bias and diagnostic delay

A notable contributor to diagnostic delay in this case was anchoring bias. The patient’s persistently elevated blood-gas bicarbonate was attributed to obstructive airway disease in the context of spirometry abnormalities; however, this attribution was not well supported by the available evidence. Inpatient CT CAP did not report emphysema or obstructive parenchymal changes to account for chronic CO₂ retention, the structural substrate most commonly underlying hypercapnia in obstructive disease. Although chronic hypercapnia can occur in obstructive disease without emphysema on CT, the degree of hypercapnia in this case was disproportionate to the pulmonary function and imaging findings. Furthermore, her spirometry obstruction demonstrated significant reversibility following bronchodilator therapy, which, whilst not excluding CO₂ retention from an obstructive aetiology entirely, rendered it a less compelling explanation for the degree of bicarbonate elevation observed, particularly in a lifelong non-smoker. In retrospect, these findings may have supported earlier consideration of an alternative mechanism for chronic hypoventilation, including neuromuscular dysfunction. 

Diagnostic delay in MND averages 10-16 months from symptom onset [1]. An elevated bicarbonate without a firmly established respiratory diagnosis, particularly in the absence of supporting structural imaging findings, should prompt systematic evaluation for neuromuscular causes of hypoventilation. Earlier pattern recognition in this case may have expedited neurological assessment and timely initiation of NIV, which is known to improve both quality of life and survival in MND-related respiratory insufficiency [7]. 

## Conclusions

This case highlights the diagnostic challenges posed by respiratory-onset MND, a rare phenotype in which neuromuscular respiratory involvement may precede overt motor signs, although the precise onset of neurological involvement could not be established in this case. Progressive unexplained dyspnoea, functional decline, and weight loss in an elderly patient should prompt consideration of a neuromuscular aetiology, particularly when the degree of respiratory compromise is disproportionate to imaging findings. 

Anchoring bias, illustrated here by the attribution of a persistently elevated bicarbonate to assumed obstructive airways disease, may have contributed to diagnostic delay. Although this retrospective association cannot be definitively established in a single case, it highlights a potentially remediable source of delay. Clinicians should maintain a broad differential when biochemical markers of chronic hypoventilation are present without a firmly established respiratory cause, and pursue early neurological assessment when the clinical picture remains unexplained.

## References

[1] O'Brien D, Shaw PJ (2024). New developments in the diagnosis and management of motor neuron disease. Br Med Bull.

[2] Masrori P, Van Damme P (2020). Amyotrophic lateral sclerosis: a clinical review. Eur J Neurol.

[3] Pinto S, Gromicho M, Oliveira Santos MO, Swash M, De Carvalho M (2023). Respiratory onset in amyotrophic lateral sclerosis: clinical features and spreading pattern. Amyotroph Lateral Scler Frontotemporal Degener.

[4] Gautier G, Verschueren A, Monnier A, Attarian S, Salort-Campana E, Pouget J (2010). ALS with respiratory onset: clinical features and effects of non-invasive ventilation on the prognosis. Amyotroph Lateral Scler.

[5] Shoesmith CL, Findlater K, Rowe A, Strong MJ (2007). Prognosis of amyotrophic lateral sclerosis with respiratory onset. J Neurol Neurosurg Psychiatry.

[6] Chan TW (2021). Presentation of motor neuron disease in a patient with weight loss and acute-on-chronic respiratory failure. BMJ Case Rep.

[7] Bourke SC, Tomlinson M, Williams TL, Bullock RE, Shaw PJ, Gibson GJ (2006). Effects of non-invasive ventilation on survival and quality of life in patients with amyotrophic lateral sclerosis: a randomised controlled trial. Lancet Neurol.

